# Three-Dimensional Finite Element Analysis of Worn Molars With Prosthetic Crowns and Onlays Made of Various Materials

**DOI:** 10.7759/cureus.30240

**Published:** 2022-10-12

**Authors:** Raafat Houdaifa, Hasan Alzoubi, Issam Jamous

**Affiliations:** 1 Department of Prosthodontics, Damascus University, Damascus, SYR; 2 Department of Pediatric Dentistry, Damascus University, Damascus, SYR

**Keywords:** 3d finite element analysis, functional forces, onlays, full crowns, parafunctional forces

## Abstract

Background/Purpose

Restoration of worn teeth represents a challenge for practitioners in terms of preserving dental tissues, achieving restoration requirements, and choosing the most appropriate material. This study aimed to evaluate the effect of both preparation and restoration type on stress distribution in modeled first mandibular molars when functional and parafunctional occlusal forces were applied.

Materials and methods

The study sample consisted of 40 three-dimensional computer models of restored lower first molars with full crowns (gold, nickel-chrome, lithium disilicate, BruxZir® zirconia, and porcelain fused to metal) and onlays (gold, nickel, chrome, lithium disilicate, and direct and indirect composites). Forces of different intensities and directions were applied, and then finite element analysis was carried out based on the von Mises equivalent stress theory to predict the failure that could occur in the restoring materials and luting cement or bonding agent.

Results

In functional forces groups, zirconia crowns showed the lowest value of the failure risk, while the highest value was in veneering porcelain with values close to the rest of the models. For onlays, gold onlays represented the best stress distribution with the lowest value of the failure risk, in contrast to the composite onlays that had the highest failure risk. In parafunctional forces groups, the preference remained for zirconia and gold crowns, as well as for metal onlays, with greater differences in the values of the failure risk.

Conclusion

Gold alloy exhibited better behavior in the stress distribution. All restorations showed similar behavior when applying functional forces; however, when applying parafunctional forces, both gold and zirconia crowns have shown the best results.

## Introduction

All prosthetics are subjected to various mechanical stresses while in the moist, temperature-changing oral environment in the presence of multiple masticatory forces. Accordingly, the clinical decision to choose the prosthesis depends on a large number of factors such as the location of the tooth, the cosmetic aspect, the patient's desire, occlusal forces, and knowledge of material properties [1].

The presence of general diseases, undesirable behavior, or bad habits that affect oral health can sometimes increase the difficulty of reaching an appropriate clinical decision. Teeth wear is an example of this as it is considered a multifactor pathogenetic mechanism that includes erosion abrasion and attrition [2].

Tooth wear is a general term that can be used to describe the loss of dental hard tissues from causes not related to caries or trauma or as a result of developmental disorders [3]. Currently, dental wear and its management pose a new challenge in dental sciences as it is becoming more and more common with time. Some epidemiological studies indicated severe dental wear with dentin exposure in about 3% of patients aged 20 years and 15% of patients aged 70 years [4].

A large number of prosthetic options are available for these patients, such as cast metallic prostheses, metal-ceramic prostheses, full ceramic prostheses, indirect veneers, and direct and indirect composite resin restorations, but there is no strong evidence in the medical literature to support the use of a specific material or method [5].

Glidewell Laboratories, Newport Beach, California, recently introduced monolithic BruxZir crowns as a cosmetic alternative to metal-ceramic crowns, with the use of a minimal preparation similar to that of cast metal crowns making them a good option with cosmetic properties [6].

To test new materials or to compare different materials, numerical methods appear to be a suitable option for assessing the distribution of stresses in the restored tooth, but the finite element method (FEM) is the most acceptable technique. The FEM is a numerical method that finds approximate solutions to the distribution of variables that are difficult to find by traditional analytical methods [7]. Therefore, the FEM will be used to study the distribution of stresses in dental prostheses of worn teeth for future clinical studies.

## Materials and methods

Study design

This is a modeled study by a three-dimensional FEM for the stress distribution in the restored worn molars with full crowns made of gold, nickel-chrome, ceramic fused to metal, ceramic reinforced with lithium disilicate, zirconia BruxZir or onlays made of gold, nickel-chrome, direct and indirect composites, and ceramic reinforced with lithium disilicate. The studied samples consisted of a three-dimensional computer model of a restored lower first molar with full crowns or onlays, and the model was represented by different restoration materials and forces of different intensities, which were applied in several directions. The total number of models studied, taking into account the restoration material and the direction of the force, was 40 models.

Each model is represented by different restoration materials: nickel-chrome, type III gold, composite resin for direct application (Filtek™, 3M ESPE, USA), composite resin for indirect application (Paradigm MZ100, 3M ESPE, USA), ceramic reinforced with lithium disilicate (IPS e.max CAD ceramics, Ivoclar Vivadent, Liechtenstein), Zirconia BruxZir (Glidewell Laboratories, USA), porcelain fused to a metal (the core of a nickel-chrome alloy), and luting cement (PANAVIA™ SA, Kuraray Dental Company, Tokyo, Japan). Forces of different intensities and directions were applied as follows. Functional forces: (1) A vertical force of 600 N is distributed on the internal and external surfaces of the buccal cusps, the central pit, and both mesial and distal margins (VF1), and (2) an oblique 45-degree force of 600 N is distributed on the external surfaces of the buccal cusps (OF1); p​​​arafunctional forces: (3) A vertical force of 1000 N is distributed on the internal and external surfaces of the buccal cusps, the central pit, and both mesial and distal margins (VF2), and (4) an oblique 45-degree force of 600 N is distributed on the external surfaces of the buccal cusps (OF2).

Building a 3D digital model of teeth and surrounding structures using SolidWorks® premium 2020 x 64 edition

Initially, a three-dimensional model of the lower molar with two roots was built, where the external shape of the mesial and distal roots was created according to the dimensions shown in Table 1.

**Table 1:** Dimensions of the mesial and distal roots of the lower molar

Unit	Mesial root					Distal root				
	Root length	Mesial-distal on furcation	Mesial-distal on apical foramen	Labial-lingual on trunk	Labial-lingual on apical foramen	Root length	Mesial-distal on furcation	Mesial-distal on apical foramen	Labial-lingual on trunk	Labial-lingual on apical foramen
mm	14	4	1.5	9	3	14	4	1.5	9	2.5

Then, two mesial canals and a distal canal were designed, taking into account that the curvature of the mesial and lingual labial canals is 28 degrees, while the distal canal is almost straight. While in the mesial-distal direction, the mesial and lingual labial canals were designed with a curvature of 21 degrees. The external shape of the mesial and distal canals was created according to the dimensions shown in Table 2.

**Table 2:** Dimensions of the mesial and distal canals of the lower molar

	Foramen size	Buccal-lingual dimensions			Mesial-distal dimensions		
		1 mm from the foramen	2 mm from the foramen	5 mm from the foramen	1 mm from the foramen	2 mm from the foramen	5 mm from the foramen
Mesial-buccal canal	0.2575	0.40	0.42	0.64	0.21	0.26	0.32
Mesial-lingual canal	0.2575	0.38	0.44	0.61	0.28	0.24	0.35
Distal canal	0.392	0.46	0.50	1.07	0.35	0.34	0.59

Then, the roots were surrounded by a ligament of 0.20 mm, and the bone was represented like a bone block and was divided into two parts, namely, cortical bone and spongy bone. The thickness of the cortical bone was 3 mm, and the thickness of the spongy bone was 15 mm. The coronal pulp was drawn; the distance from the floor of the pulp chamber to the furcation area was 3 mm, and the height of the pulp chamber to the level of the pulpal horn was 2 mm. Then, the coronal section of the tooth was drawn according to the median dimensions of the crown of the lower first molar as shown in Table 3. The thicknesses of the enamel and dentin are, on average, 1.8 mm and 3 mm in the occlusal direction and 1.2 and 2.5 mm in the direction of mesial-distal walls, respectively, and as the samples are severe tooth wear, the clinical length of the remaining crown was 5.5 mm.

**Table 3:** Median dimensions of the crown of the first lower molar in mm

Occlusal-cervical dimensions	Buccal-lingual dimensions	Buccal-lingual dimensions on cervical	Mesial-distal dimensions	Mesial-distal dimensions on cervical
7.5	10.5	9	11	9

Preparation dimensions

**Figure 1:**
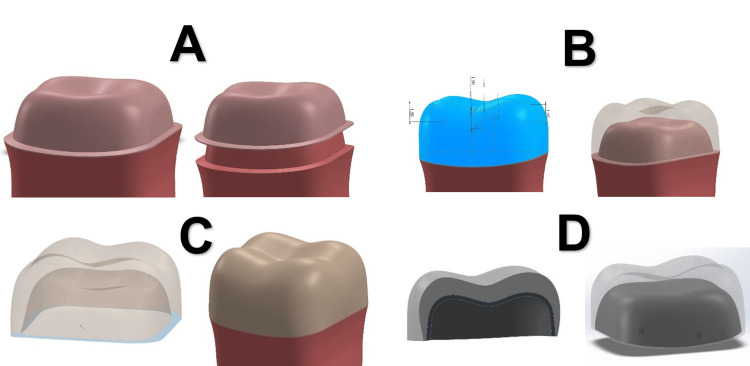
3D models of the full crown: (A) cement layer, (B) dimension of the full crown, (C) full crown, and (D) porcelain fused to metal crown

**Figure 2:**
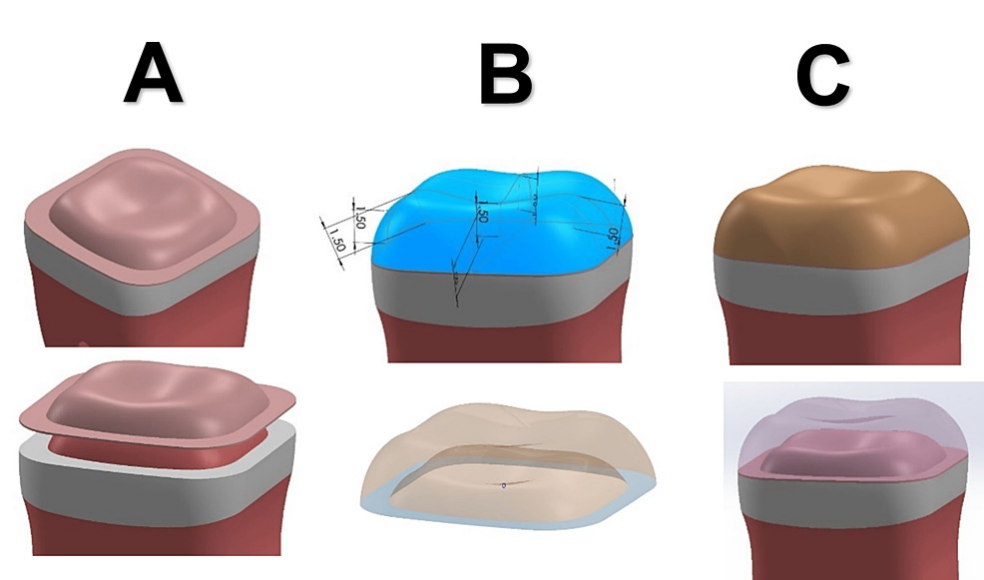
3D models of the onlays: (A) cement layer, (B) dimension of onlay, and (C) onlay

In the model prepared to receive a full crown, the abutment length was 3.5 mm, and the axial walls tilted 12° with a chamfer of 0.5 mm. In the model prepared to receive onlays, the abutment length was 3.5 mm with an occlusal shoulder of 0.7 mm and a layer of enamel with a thickness of 1.2 mm median on the axial walls. In the crowns and onlays, the luting cement was drawn initially with a thickness of 100 microns based on the anatomical shape of the prepared tooth; except for the prepared tooth to receive direct composite, the adhesion layer with a thickness of 10 microns was represented, then the crowns and onlays were drawn with a thickness of 1.5 mm on average so that it restores the anatomical shape of cusps and axial walls, and the metal-ceramic crown was drawn so that the thickness of the metal was 0.3 mm and the covered ceramic was 1.2 mm (Figures 1, 2).

Powershape program

Each component of the design was individually imported into the program, and errors, if any, such as overlaps and gaps were corrected by the Solid Doctor command, and then each model was exported separately in Parasolid format with the suffix (x-t).

Analysis System (Ansys) program

Initially, a special library for the materials included in the study was created by engineering data from the components' systems, but later, the library was modified from engineering data sources, and the materials and their properties were inserted (Young's modulus and Poisson's modulus) as shown in Table 4. All components were considered linear elastic, isotropic, and homogeneous.

**Table 4:** The characteristics of the studied materials

Material	Young's modulus	Poisson's modulus	Reference No.
Enamel	84.1 x 10^3^	0.33	[8]
Dentin	18.6 x 10^3^	0.32	
Pulp	0.002 x 10^3^	0.45	
Ligament	0.069 x 10^3^	0.45	
Cortical bone	13.7 x 10^3^	0.30	
Spongy bone	1.37 x 10^3^	0.30	
Gold	91 x 10^3^	0.33	
Nickel-chrome	205 x 10^3^	0.33	
Direct composite	12.7 x 10^3^	0.35	[9]
Indirect composite	16 x 10^3^	0.24	[10]
Ceramic reinforced with lithium disilicate	83.5 x 10^3^	0.21	[11]
Porcelain fused to metal	68.9 x 10^3^	0.28	[12]
Zirconia	210 x 10^3^	0.30	[12]
Luting cement	12 x 10^3^	0.24	[13]
Adhesive layer	3 x 10^3^	0.30	[14]

**Figure 3:**
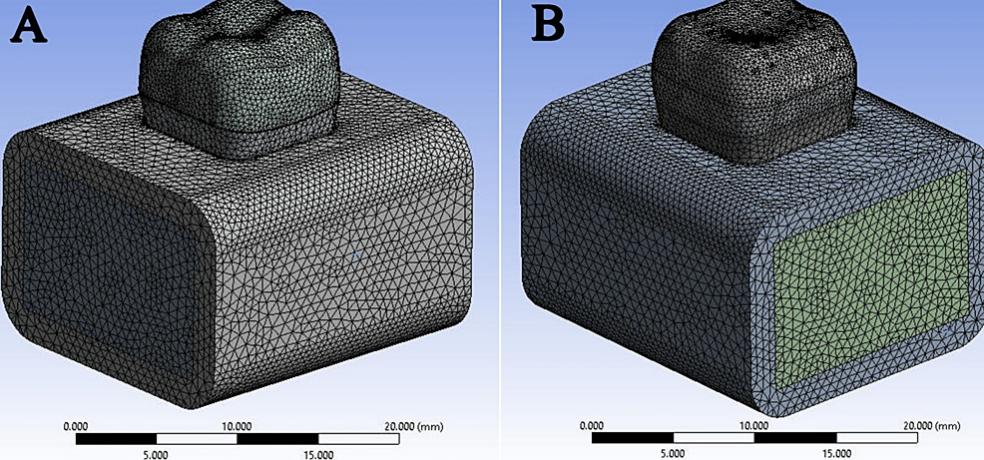
Meshing process: (A) full crowns and (B) onlay

The work was followed up by the Ansys Mechanical Enterprise through the Model command, and then the interfaces between the different parts were checked as they were all bonded style without voids or gaps. The 3D model has meshed into small regular geometric shapes called tetrahedral elements through a Mesh process, the quadratic pattern of the elements was selected, the default size was adopted, and some parts such as restoration, dentin, and enamel were refined. The average number of nodes and elements in the study models is shown in Table 5 and Figure 3.

**Table 5:** The average number of nodes and elements in the study models

Model	Nodes	Models
Full crown	430,087	252,830
Ceramic fused to metal	429,516	247,757

Determine the area of ​​fixation or support, the amount of force applied, its direction, and places of application as follows. Support: Select the lower surface of the cortical bone as the fixed support area, and approve it as the surface farthest from the place of application of force to avoid any interference or error in the result. Force: The forces were applied in different directions as mentioned previously.

Subsequently, the type of analysis to be performed was chosen, and an equivalent stress (von Mises) analysis was conducted in this study to find out the distribution of stresses in the components of the models, starting with solving and obtaining the results. Then, each of the following equations was applied: $SC = \frac{Sp}{aS}$, where SC is the stress concentration; Sp is the stress peak, which is the highest stress value in the studied area, and aS is the average value of the stresses in the studied area. $FR = \frac{Sp}{Strength}$, where FR is the failure risk, Sp is the stress peak, which is the highest stress value in the studied area, and strength is the resistance of the studied material [15].

## Results

The concentration of stresses in all models was observed in the areas of force application and in the cervical region, especially the buccal, mesial, and distal regions, regardless of the direction of the applied force. On the other hand, the parafunctional forces led to an increase in the stress values ​​and their concentration in all models.

**Figure 4:**
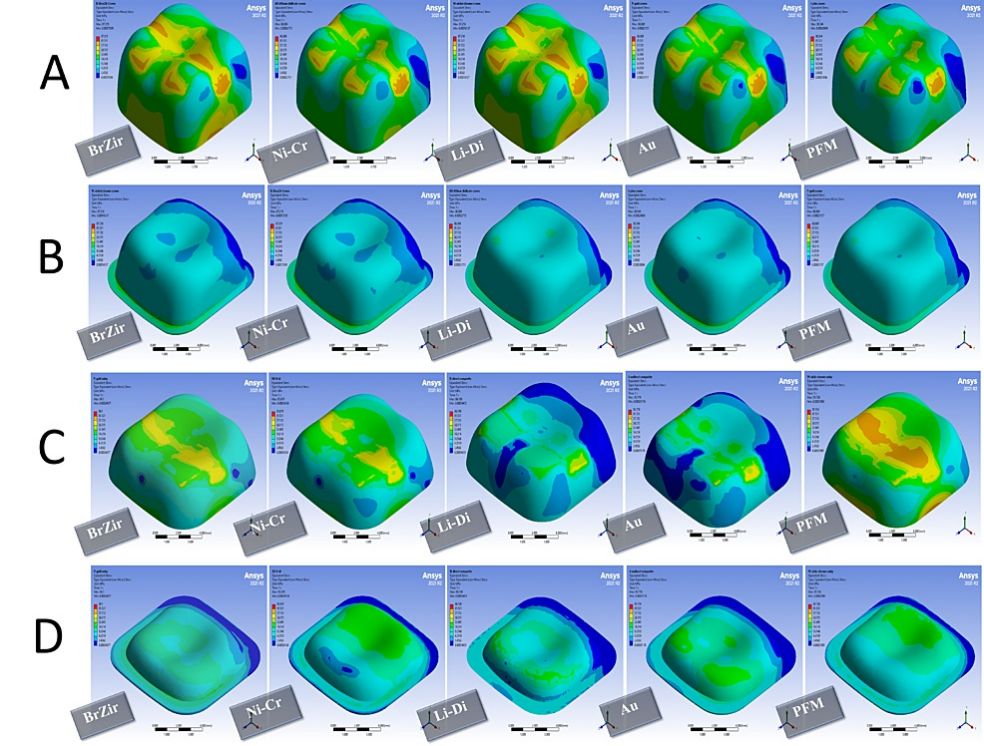
Stress distribution in 600 N vertical forces: (A) full crown restorations, (B) full crown luting cement, (C) onlays restorations, and (D) onlays luting cement BrZir: BruxZir; AU: Gold alloy; Ni-Cr: Nickel-chrome; Li-Di: Lithium disilicate; PFM: Porcelain fused to metal.

**Figure 5:**
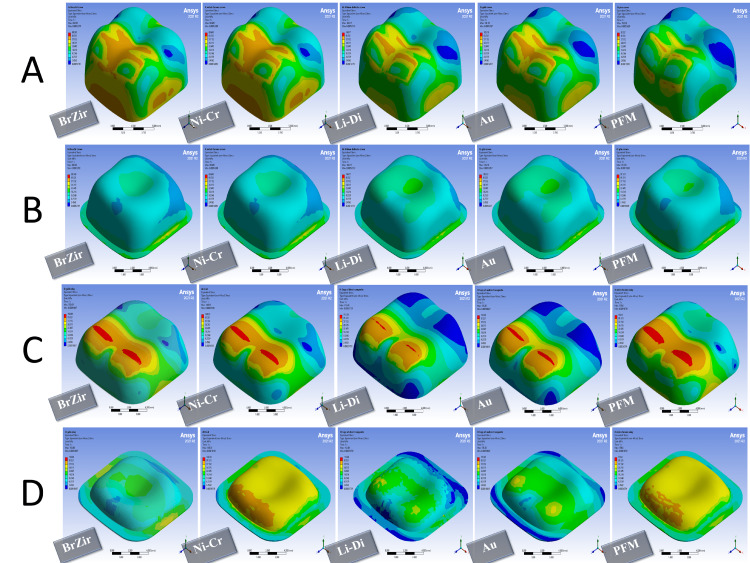
Stress distribution in 600 N oblique forces: (A) full crown restorations, (B) full crown luting cement, (C) onlays restorations, and (D) onlays luting cement BrZir: BruxZir; AU: Gold alloy; Ni-Cr: Nickel-chrome; Li-Di: Lithium disilicate; PFM: Porcelain fused to metal.

When the functional force was applied, in full crown groups, it was noted that the best distribution of stresses and the lowest value of the failure risk were in zirconia crowns, then in metal and gold crowns, while the highest value of the failure risk was for veneering porcelain, then in lithium disilicate crown. As for luting cement, both lithium disilicate and gold crowns showed the best stress distribution model with the lowest value of the failure risk, while the highest value of the failure risk was in PFM crowns, then metal and zirconia crowns (Figures 4, 5 and Table 6). In onlay groups, the best distribution of stresses was observed in the gold onlays with the lowest failure risk, regardless of the direction of the force, followed by nickel-chrome onlays, and the highest failure risk values were for the composite resin onlays. For the luting cement used and the bonding layer, it was noted that the failure risk was the highest in indirect composite onlays (Figures 4, 5 and Table 6).

**Table 6:** Stress concentration and failure risk in VF1 and OF1 groups VF1: Vertical force 600 N; OF1: Oblique force 600 N; SC: Stress concentration; FR: Failure risk; AU: Gold alloy; Ni-Cr: Nickel-chrome; Li-Di: Lithium disilicate; VP: Veneering porcelain; PFM: Porcelain fused to metal; DC: Direct composite; IND: Indirect composite.

Loading	Prosthesis type	Material		Tensile strength	Prosthesis				Tensile strength	Cement layer			
					Stress peak	Average stress	SC	FR		Stress peak	Average stress	SC	FR
VF1	Crown	BrZir		745 [10]	55.495	14.1	2.94	0.07	114.1 [16]	18.376	5.1561	3.56	1.30
		AU		483 [10]	53.495	13.9	3.84	0.11		15.917	5.6023	2.84	1.12
		Ni-Cr		559 [17]	55.388	18.603	2.97	0.09		18.355	5.1703	3.55	1.30
		Li-Di		173 [18]	53.689	13.796	3.89	0.31		15.454	5.5893	2.76	1.09
		PFM	VP	69 [12]	48.767	10.648	4.57	0.70		19.23	5.8348	3.29	1.36
			Ni-Cr	559	62.094	27.375	2.68	0.11					
	Onlays	DC		84 [18]	33.627	20 [13]	5.35	0.40	20 [19]	20.619	5.9912	3.44	1.03
		IND		54.4 [10]	32.63	14.1	4.50	0.59	14.1	20.148	6.7801	2.97	1.42
		AU		483	38.263	12.903	2.96	0.06		16.044	6.39	2.51	1.13
		Ni-Cr		559	59.054	18.783	3.14	0.10		17.718	8.5366	2.07	1.25
		Li-Di		173	40.292	12.622	3.19	0.23		18.64	8.7069	2.14	1.32
OF1	Crown	BrZir		745	75.776	14.1	3.21	0.10	14.1	30.791	7.4243	4.14	2.18
		AU		483	68.64	18.146	3.78	0.14		26.62	8.1007	3.28	1.88
		Ni-Cr		559	76.091	23.374	3.25	0.13		30.625	7.4536	4.10	2.17
		Li-Di		173	67.327	17.786	3.78	0.38		26.609	8.0642	3.29	1.88
		PFM	VP	69	47.602	12.076	3.9	0.68		28.296	8.7284	3.24	2.00
			Ni-Cr	559	72.44	36.591	1.97	0.12					
	Onlays	DC		84 [20]	112.04	20	8.77	1.33	20	35.393	8.5597	4.13	1.76
		IND		54.4 [10]	135.03	14.1	8.57	2.48	14.1	29.168	9.9056	2.94	2.06
		AU		483	150.89	21.933	6.87	0.31		30.307	10.238	2.96	2.14
		Ni-Cr		559	159.6	25.457	6.26	0.28		51.061	24.081	2.12	3.62
		Li-Di		173	149.33	21.121	7.07	0.86		55.447	23.602	2.34	3.93

**Figure 6:**
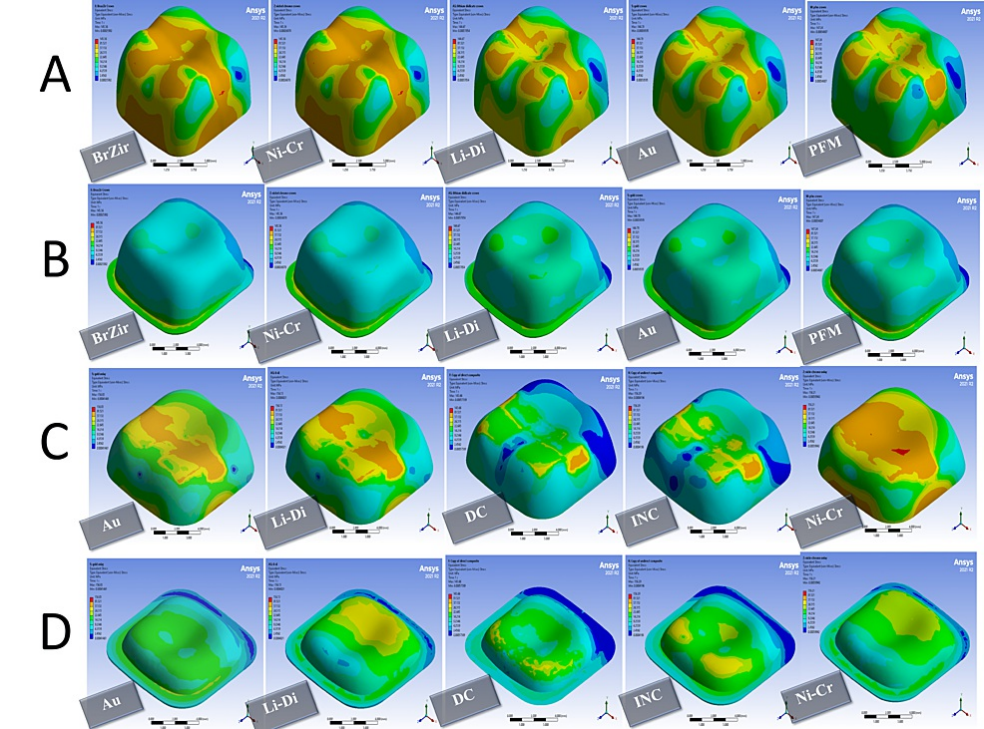
Stress distribution in 1000 N vertical forces: (A) full crown restorations, (B) full crown luting cement, (C) onlays restorations, and (D) onlays luting cement BrZir: BruxZir; AU: Gold alloy; Ni-Cr: Nickel-chrome; Li-Di: Lithium disilicate; PFM: Porcelain fused to metal; DC: Direct composite; INC: Indirect composite.

**Figure 7:**
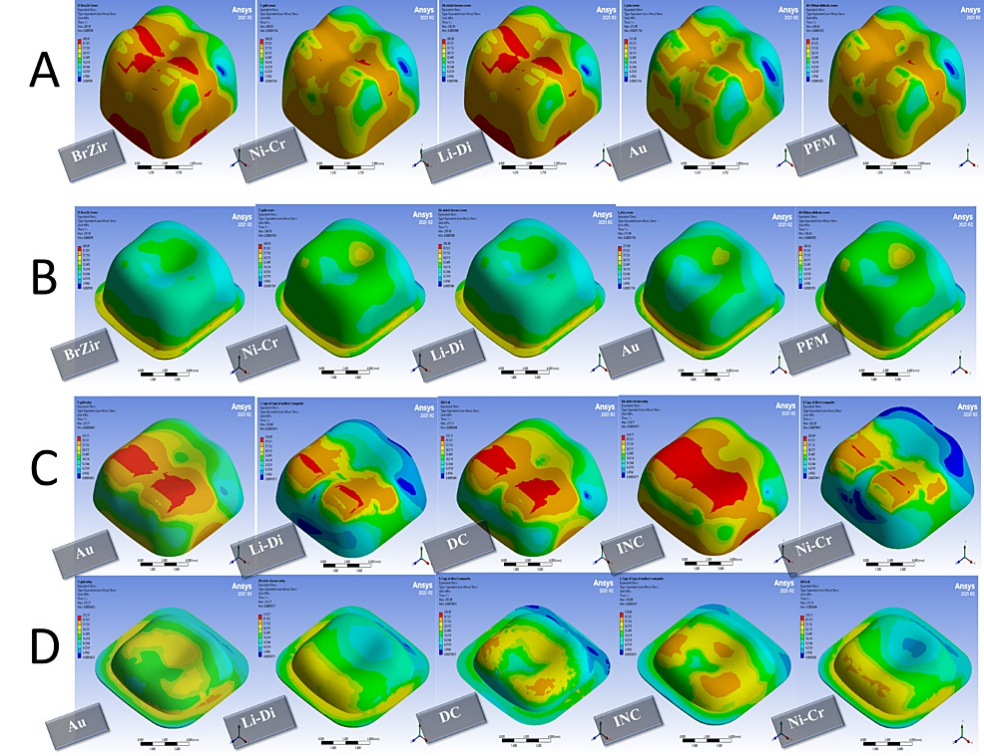
Stress distribution in 1000 N vertical forces with 600 N oblique forces: (A) full crown restorations, (B) full crown luting cement, (C) onlays restorations, and (D) onlays luting cement BrZir: BruxZir; AU: Gold alloy; Ni-Cr: Nickel-chrome; Li-Di: Lithium disilicate; PFM: Porcelain fused to metal; DC: Direct composite; INC: Indirect composite.

When a parafunctional force was applied, in full crown groups, both zirconia and gold crowns showed the lowest value of the failure risk at the level of restoration. The highest failure risk was at the veneering porcelain in PFM crowns and then lithium disilicate. For luting cement, gold and lithium disilicate crowns had the preference in terms of distribution stresses with the lowest value of the failure risk, and the highest value of the failure risk was in PFM crowns (Figures 6, 7 and Table 7) In onlay groups, the composite resin onlays were the highest failure risk at the level of restoration with the best performance for the gold and nickel-chrome onlays. For luting cement, indirect composite onlays were the highest failure risk (Figures 6, 7 and Table 7).

**Table 7:** Stress concentration and failure risk in VF2 and OF2 groups VF2: Vertical force 1000 N; OF2: Vertical force 1000 with oblique force 600 N; SC: Stress concentration; FR: Failure risk; AU: Gold alloy; Ni-Cr: Nickel-chrome; Li-Di: Lithium disilicate; VP: Veneering porcelain; PFM: Porcelain fused to metal; DC: Direct composite; IND: Indirect composite.

Loading	Prosthesis type	Material		Tensile strength	Prosthesis				Tensile strength	Cement layer			
					Stress peak	Average stress	SC	FR		Stress peak	Average stress	SC	FR
VF2	Crown	BrZir		745	92.492	31.371	2.94	0.12	14.1	30.626	8.5935	3.56	2.17
		AU		483	89.159	23.166	3.84	0.18		26.528	9.3372	2.84	1.88
		Ni-Cr		559	92.314	31.005	2.97	0.16		30.592	8.6171	3.55	2.16
		Li-Di		173	89.482	22.994	3.89	0.51		25.756	9.3156	2.76	1.82
		PFM	VP	69	81.279	17.747	4.57	1.17		32.05	9.7247	3.29	2.27
			Ni-Cr	559	103.49	45.624	2.26	0.18					
	Onlays	DC		84	56.045	10.464	5.35	0.66	20	34.365	9.9853	3.44	1.71
		IND		54.4	54.383	12.081	4.50	0.99	14.1	33.581	11.3	2.97	2.38
		AU		483	63.771	21.505	2.96	0.13		26.74	10.65	2.51	1.89
		Ni-Cr		559	98.423	31.306	3.14	0.17		29.529	14.228	2.07	2.09
		Li-Di		173	67.153	21.036	3.19	0.38		31.066	14.511	2.14	2.20
OF2	Crown	BrZir		745	123.98	46.702	2.65	0.16	14.1	41.239	13.586	3.03	2.92
		AU		483	91.992	34.542	2.66	0.19		36.669	14.75	2.48	2.60
		Ni-Cr		559	121.31	46.22	2.62	0.21		41.181	13.638	3.01	2.92
		Li-Di		173	91.75	34.00	2.69	0.53		35.678	14.647	2.43	2.53
		PFM	VP	69	82.1	24.312	3.37	1.18		47.563	15.582	3.05	3.37
			Ni-Cr	559	143.13	67.776	2.11	0.25					
	Onlays	DC		84	109.85	18.293	6.00	1.30	20	53.245	16.288	3.26	2.66
		IND		54.4	135.48	22.009	6.15	2.49	14.1	53.438	18.401	2.90	3.78
		AU		483	170.13	36.853	4.61	0.35		43.371	18.558	2.33	3.07
		Ni-Cr		559	196.4	50.212	3.91	0.35		40.391	17.794	2.26	2.86
		Li-Di		173	164.18	35.942	4.56	0.94		48.021	18.033	2.66	3.40

## Discussion

Worn teeth are one of the great challenges facing practitioners, especially with their more and more common occurrence, and their management poses many difficulties. The significant loss of dental tissue dictates the need to preserve the rest of the tooth, especially the enamel layer, and maintain its vitality [21]. The literature lacks strong evidence to support the use of a specific material for the management of worn teeth; although a large number of materials are available [2,5], type III gold and nickel-chrome alloys have traditionally been used to restore worn teeth, and development of types of cement modified with chemical resins made it possible to apply them with conservative minimal preparations (inlays and onlays), in addition to applying them as crowns [22]. The association of bruxism with attrition poses another challenge; the occlusal forces to which restorations are exposed, regardless of their type and material, are non-functional forces and differ in intensity and direction from the natural occlusal forces, which usually manifest with two non-functional behaviors such as clenching and grinding [23].

The finite element analysis is an effective method for analyzing the mechanical behavior of complex structures as it helps in evaluating the different stresses generated in the oral cavity and studying the effect of the design of restorations and various restorative materials on the distribution of stresses [7,24]. It is also useful in understanding the failure patterns in general and the areas from which failure is expected to begin, thus helping to explain the causes of clinically observed failure as well as predicting the clinical performance of restorative materials [25]. The study was carried out using a three-dimensional model of a lower first molar as most studies indicate that the occlusal surfaces of the lower molars are most susceptible to abrasion and are the highest in the molar region [19].

The length of the clinical crown of the first molar was 7.5 mm on average [26], and given that the value of the wear was 2 mm, the length of the entire tooth with the restoration was 5.5 mm. Therefore, an average thickness of 1.5-2 mm was adopted for all restorations as it was considered that the restorations were made to the minimum or acceptable thickness according to previous studies [27,28]. The same applies to the ceramic fused to the metal crown, where 0.3 mm was adopted for the metal and 1.2 mm was adopted for the veneering porcelain [29]. For crowns, the chamfer was adopted as a type of preparation with a width of 0.5 mm with a tilt of the axial walls of 12 degrees without any residual enamel [29]. For onlays, an occlusal chamfer with a width of 0.7 mm was designed within the enamel layer as described by Mehta et al. [30].

In functional forces, for crowns, it was noted that the best distribution of stresses and the lowest value of the failure risk was in the zirconia crowns, then in the metal and the gold crowns. The highest value of the failure risk was for the veneering porcelain in the metal-ceramic and lithium disilicate, regardless of the direction of the force. This may be explained by the high modulus of elasticity of these materials and their ability to withstand different forces, which makes failure at the level of the restoration material unlikely. These results agreed with the results of Ausiello et al. [14]. As for luting cement, both lithium disilicate and gold crowns showed the best stress distribution with the lowest value of the failure risk. The highest value of the failure risk was in PFM and zirconia crowns, which is consistent with the studies that show restoration dislocation is the most common complication in zirconia crowns [31].

In onlays, the best distribution of stress was observed in the gold onlays with the lowest failure risk, regardless of the direction of the force, followed by both the metal and lithium disilicate onlays. The highest values ​​of the failure risk were for the composite resin onlays, and this is explained by the high value of the stress concentration in them, that is, the stress concentration increased in specific areas in the restoration with a small cross-section, making it more susceptible to fracture. The results of this study differed from the results of the study of D’souza and Aras [8], and this difference may be attributed to relying only on the color maps without relying on the stress concentration rate, which may give ambiguous results. As for the luting cement used for these onlays and the bonding layer of the direct composite resin, it was noted that the failure risk was highest in each of the lithium disilicate and metal onlays, and although the stress distribution in the bonding layer was not the best, it had the lowest value of the failure risk, which is explained by the high resistance of the bonding layer compared to resin cement.

When applying parafunctional force, as for the crowns, both zirconia crowns and gold crowns showed the lowest value of the failure risk that could be obtained at the level of restoration. Lithium disilicate and veneering porcelain were the closest to failure, which is consistent with the studies that indicated veneering porcelain fracture is primarily responsible for the failure of ceramic fused to metal crowns [12]. As for luting cement, the gold and the lithium disilicate crowns had the advantage of the distribution of stresses. The most susceptible to loosening was PFM and zirconia crowns. As for onlays, the composite resin was the most likely to fail at the level of restoration with the best performance for the gold and metal cast onlays. The indirect composite onlays were the most likely to fail at the level of the bonding layer.

This study faced some limitations due to the nature of this method used to simplify the engineering processes. All materials and structures were represented as linearly flexible, symmetric, and homogeneous in properties. Although the force was represented in different directions and intensities to simulate the clinical reality as much as possible, this study was done in the manner of static structures, so it does not completely match the clinical reality in terms of occlusal complexity and lower jaw movements.

## Conclusions

The stresses generated within onlays were significantly higher than the stresses generated within full crowns. The ratio of the distribution of stresses in the components of the tooth was related to the elasticity modulus of the restorations used, and the ratio of stresses transmitted to the tooth decreased with an increase in the elasticity modulus of the used materials.

Cast gold, whether used as a full crown or onlays, showed the best performance in terms of stress distribution for the restoration itself and the luting cement. BruxZir crowns showed the lowest value of the failure rate, but they showed high values ​​of the failure rate for the restoration loosening.
